# Comparison of cone bioassay estimates at two laboratories with different *Anopheles* mosquitoes for quality assurance of pyrethroid insecticide-treated nets

**DOI:** 10.1186/s12936-022-04217-3

**Published:** 2022-07-07

**Authors:** Stephen G. Mbwambo, Nakei Bubun, Emmanuel Mbuba, Jason Moore, Kasiani Mbina, Dismas Kamande, Moses Laman, Emmanuel Mpolya, Olukayode G. Odufuwa, Tim Freeman, Stephan Karl, Sarah J. Moore

**Affiliations:** 1 Vector Control Product Testing Unit (VCPTU), Environmental Health and Ecological Science Department, Ifakara Health Institute, Bagamoyo, Tanzania; 2 Nelson Mandela Africa Institution of Science and Technology, Arusha, Tanzania; 3 Sokoine RRH, Ministry of Health, Lindi, Tanzania; 4 Regional Health Management Team, P.O Box 1011, Lindi, Tanzania; 5 Vector Borne Disease Unit, PNG Institute of Medical Research, Madang Province 511, P.O Box 378, Madang, Papua New Guinea; 6 University of Basel, Basel, Switzerland; 7 Vector Biology Unit, Department of Epidemiology and Public Health, Swiss Tropical and Public Health Institute (Swiss TPH, Allschwil, Kreuzstrasse 2, 4123 , Basel, Switzerland; 8 MRC International Statistics and Epidemiology Group, London School of Hygiene and Tropical Medicine, Keppel Street, London, WC1E 7HT UK; 9 Rotarian Against Malaria, P.O Box 3686, Boroko, NCD 111 Papua New Guinea; 10 Australian Institute of Tropical Health and Medicine, James Cook University, 1/14-88 McGregor Road, Smithfield, QLD 4870 Australia

**Keywords:** Bioefficacy, Bioassay, Cone bioassay, Tunnel test, Insecticide treated nets, ITN, Long lasting insecticidal nets, LLIN, Pyrethroid, Mosquito, *Anopheles*, Malaria, Quality assurance

## Abstract

**Background:**

Quality assurance (QA) of insecticide-treated nets (ITNs) delivered to malaria-endemic countries is conducted by measuring physiochemical parameters, but not bioefficacy against malaria mosquitoes. This study explored utility of cone bioassays for pre-delivery QA of pyrethroid ITNs to test the assumption that cone bioassays are consistent across locations, mosquito strains, and laboratories.

**Methods:**

Double-blinded bioassays were conducted on twenty unused pyrethroid ITNs of 4 brands (100 nets, 5 subsamples per net) that had been delivered for mass distribution in Papua New Guinea (PNG) having passed predelivery inspections. Cone bioassays were performed on the same net pieces following World Health Organization (WHO) guidelines at the PNG Institute of Medical Research (PNGIMR) using pyrethroid susceptible *Anopheles farauti *sensu stricto (*s.s*.) and at Ifakara Health Institute (IHI), Tanzania using pyrethroid susceptible *Anopheles gambiae s.s*. Additionally, WHO tunnel tests were conducted at IHI on ITNs that did not meet cone bioefficacy thresholds. Results from IHI and PNGIMR were compared using Spearman’s Rank correlation, Bland–Altman (BA) analysis and analysis of agreement. Literature review on the use of cone bioassays for unused pyrethroid ITNs testing was conducted.

**Results:**

In cone bioassays, 13/20 nets (65%) at IHI and 8/20 (40%) at PNGIMR met WHO bioefficacy criteria. All nets met WHO bioefficacy criteria on combined cone/tunnel tests at IHI. Results from IHI and PNGIMR correlated on 60-min knockdown (KD60) (r_s_ = 0.6,*p* = 0.002,n = 20) and 24-h mortality (M24) (r_s_ = 0.9,*p* < 0.0001,n = 20) but BA showed systematic bias between the results. Of the 5 nets with discrepant result between IHI and PNGIMR, three had confidence intervals overlapping the 80% mortality threshold, with averages within 1–3% of the threshold. Including these as a pass, the agreement between the results to predict ITN failure was good with kappa = 0.79 (0.53–1.00) and 90% accuracy.

**Conclusions:**

Based on these study findings, the WHO cone bioassay is a reproducible bioassay for ITNs with > 80% M24, and for all ITNs provided inherent stochastic variation and systematic bias are accounted for. The literature review confirms that WHO cone bioassay bioefficacy criteria have been previously achieved by all pyrethroid ITNs (unwashed), without the need for additional tunnel tests. The 80% M24 threshold remains the most reliable indicator of pyrethroid ITN quality using pyrethroid susceptible mosquitoes. In the absence of alternative tests, cone bioassays could be used as part of pre-delivery QA.

**Supplementary Information:**

The online version contains supplementary material available at 10.1186/s12936-022-04217-3.

## Background

Pyrethroid insecticide-treated nets (ITNs) are among the recommended public health interventions for control of malaria vectors [1] and are estimated to have prevented more than 450 million malaria cases in Africa between 2000 and 2015 [2]. While insecticide resistance [3] and mosquito behavioural changes [4] are factors contributing to the reduction of the effectiveness of pyrethroid ITNs, they can still provide a high degree of protection [5], especially in areas where *Anopheles* mosquitoes are still susceptible to pyrethroids like in Papua New Guinea (PNG) [3, 6].

It is important to deliver effective ITNs to protect those at risk against mosquito bites and malaria. To guarantee the effectiveness of ITNs distributed in malaria-endemic countries, it is necessary to conduct independent pre-delivery quality assurance (QA) and post-delivery operational monitoring of ITN quality [7]. Pre-delivery, ITN product specifications are checked including ITN insecticide content. Post-delivery, ITN insecticide content, bioefficacy, physical integrity, and ITN survivorship are metrics used for ITN quality monitoring [8]. Bioefficacy is a measurement of the ability of the ITN product to induce mortality, knockdown (sublethal incapacitation) or prevent blood feeding of mosquitoes under laboratory conditions. Minimum bioefficacy thresholds for laboratory assays [8], have been set at a level measured in experimental hut trials [9] that corresponded with malaria control, estimated by clinical trials conducted in Africa when mosquito vectors were still susceptible to pyrethroids [10]. Therefore, mosquito mortality benchmarks in place for ITNs are not only aimed to ensure personal protection to the user, but also that ITNs kill sufficient mosquitoes to provide community protection [11].

Pyrethroid ITN bioefficacy is evaluated experimentally under laboratory conditions with susceptible malaria vectors using cone bioassay and tunnel tests [8]. Bioefficacy evaluations provide reassurance of likely impact against susceptible vectors [8, 12–14]. New or unused pyrethroid ITNs should meet World Health Organization (WHO) standard bioefficacy criteria, i.e., ≥ 95% mosquitoes knockdown at 60-min (KD60) and/or ≥ 80% mortality at 24-h (M24) for cone bioassays [8]. It has been shown by many studies that new or unused pyrethroid ITNs generally exhibit 100% for both or either of these bioefficacy endpoint(s) [15–26]. The utility of cone bioassays is that they can estimate small variations in insecticide [18] and bioefficacy [27] that can inform the effectiveness of the intervention. For pyrethroid ITNs unable to meet cone bioefficacy criteria, a second evaluation is conducted, using the WHO tunnel test that is designed for the evaluation of ITNs treated with insecticides that have an excito-repellent mode-of-action e.g. permethrin or etofenprox [28]. However, in reality tunnel tests are used for all ITNs regardless of the mode of action of the active ingredient. The performance thresholds for WHO tunnel tests are to induce ≥ 90% blood feeding inhibition (BFI) and/or ≥ 80% M24.

Physiochemical tests are currently used for ITN QA [29] on the assumption that product performance is predictable based on the product specifications measured in predelivery inspections. Available evidence indicates that the vast majority of ITNs are likely to contain sufficient insecticide when they are delivered to households [30]. While this is encouraging, it should be remembered that predelivery inspections measure the total chemical content of the net yarn, while mosquitoes landing on the netting are exposed only to the insecticide present on the surface. The bioefficacy endpoints of KD60 or M24 are sensitive to small changes in insecticide surface concentration, which can be different between, and sometimes within, products and can be subject to change in particular when ITNs are exposed to heat [31, 32]. It has been shown that total insecticide content does not always correlate with bioefficacy [33].

Differences in ITN bioefficacy may be due to variations in spatial presentation and/or distribution of active ingredient within the netting, or the surface treatment as part of the manufacturing process. ITNs are manufactured from polyester or polyethylene, and careful product design and quality controlled manufacturing is required to ensure adequate bioavailability of active ingredient over the life of the product [31]. It is generally agreed that a validated, low-cost, easy-to-implement laboratory methodology for assessing surface AI content is urgently needed for QA [30]. However, chemical assays of surface concentration, such as the cyanopyrethroid field test and chemical tests such as high performance liquid chromatography and gas chromatography with mass spectrometry have not yet been found to correspond well to bioefficacy results [34].

To ensure successful malaria control, it is necessary to confirm bioefficacy of ITNs in pre-delivery inspections and/or prior to distribution. The cone bioassay remains the standard for determining pyrethroid ITN bioefficacy [8, 35], as it is a simple and cost-effective methodology [21]. Using cone bioassays to routinely evaluate mosquito nets before distribution was suggested and used for quality control of conventionally treated nets [36, 37] and recommended in the World Health Organization Pesticide Evaluation Scheme (WHOPES) tenth meeting [35]. However, the WHOPES eleventh meeting report concluded that “WHO standard bioassays cannot be used throughout the world for quality control purposes, so physicochemical tests must be used instead” [13]. A recent ‘Landscaping of ITN Bioefficacy Report for The Global Fund’ recommended a bioefficacy test for ITN quality monitoring both pre-and post-shipment [30]. Even so, there is some debate about variability of cone bioassays results when different *Anopheles* species are used. A robust test should ideally reveal reproducible bioefficacy results at different testing facilities on the same net samples against various *Anopheles* strains especially with similar pyrethroid susceptibility levels. Not much is known about whether cone bioassay results are subject to systematic bias depending on the *Anopheles* species used.

This study explored utility of cone bioassays for pre-delivery QA in two test facilities using different *Anopheles* species to test if cone bioassays are consistent and reproducible across locations, mosquito strains, and laboratories. A literature review on the utility of cone bioassays for unused pyrethroid ITNs testing was also conducted to gather additional information on average bioefficacy values measured for new or unused pyrethroid ITNs using the cone bioassay.

## Methods

### Study design

A double-blinded comparison of pyrethroid ITN bioefficacy as measured by WHO cone bioassay was conducted in two testing facilities. Twenty unused pyrethroid ITNs of 4 brands (100 subsamples, i.e., 5 subsamples per net) that had passed predelivery inspections were assessed under laboratory conditions following WHO guidelines [8]. ITN subsamples were first evaluated using WHO cone bioassays and those that did not meet the WHO cone bioassay performance criteria (≥ 95% KD60 or ≥ 80% 24-h mortality) were tested using the WHO tunnel test at IHI following standard procedures [8].

### Testing facilities

The experiments were conducted at the Vector-borne Diseases Unit (VBDU) of the Papua New Guinea Institute of Medical Research (PNGIMR) and the Vector Control Product Testing Unit (VCPTU) of the Ifakara Health Institute (IHI) in Tanzania that is Good Laboratory Practice (GLP) accredited, South African National Accreditation System (SANAS) G0033 [38].

## Description of tested products

Five products (rectangular nets) were included in the study (Additional file 1).

i. PermaNet^®^ 2.0, a blue multi-filament polyester, 75 denier coated with 1.8 g/kg (55 mg/m^2^) deltamethrin and manufactured in 2019 by Vestergaard in Vietnam;
ii. PermaNet^®^ 2.0, a yellow multi-filament polyester net, 75 denier coated with 1.8 g/kg (55 mg/m^2^) deltamethrin and manufactured in 2012 (manufacture location not given on label);
iii. Interceptor^®^, a blue multi-filament polyester net, 100 denier coated with 5 g/kg (200 mg/m^2^) alpha-cypermethrin and manufactured in 2020 by BASF in Thailand;
iv. SafeNet^®^, a blue multifilament polyester net, 100 denier coated with 5 g/kg (200 mg/m^2^) alpha-cypermethrin (manufactured in 2019 and 2020, manufacture location not given on label);
v. Yorkool^®^, a blue multifilament polyester net, 75 denier coated with 1.8 g/kg (55 mg/m^2^) deltamethrin and manufactured in 2019 by Tianjin Yorkool International Trading Company Limited in China.

Negative control net: untreated SafiNet^®^ manufactured by A to Z textile mills in Tanzania and untreated Baomei^®^ net manufactured in China were used in IHI and PNGIMR, respectively.

## Net origin and storage condition

The PermaNet^®^ 2.0 manufactured in 2012 (PermaNet^®^2012) nets were distributed in the year 2012 through the mass distribution campaign in PNG. These ITNs in unopened packaging were stored under tropical temperature and humidity in a storeroom of the Madang Provincial Health Authority between 2012 and 2018. The nets were transferred to a PNGIMR store in 2018 and kept at around 27 °C. Other ITNs i.e. the PermaNet^®^ 2.0 manufactured in 2019 (PermaNet^®^2019), the Interceptor^®^, the SafeNet^®^ and the Yorkool^®^ were collected from shipping containers immediately upon arrival in PNG and prior to distribution, and stored a PNGIMR store room at around 27 °C.

## Net subsamples preparation and coding

**Fig. 1:**
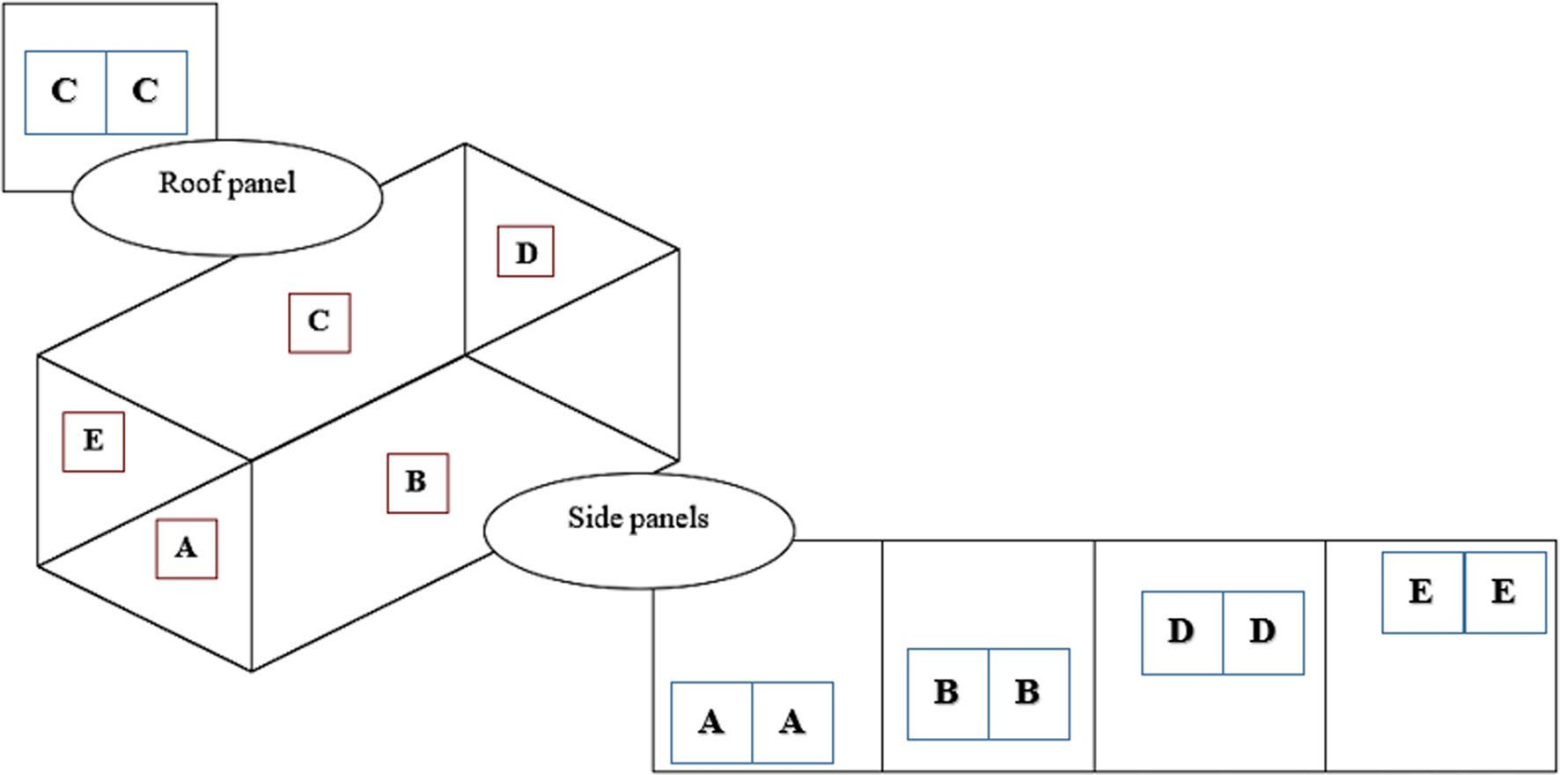
Rectangular whole net with five sides; net piece samples were cut from bottom side (**A**), middle side A (**B**), roof (**C**), middle side B (**D**) and top side (**E**)

The sampled ITNs were labelled serially from 001 to 020 at PNGIMR. From these nets, five net piece samples (25 cm × 25 cm) were cut. Samples were cut from positions 1 to 5 as shown in (Fig. 1) [8]. The nets were tested in PNG and then the same pieces were sent to IHI for testing. Thus, one hundred net pieces were each tested in PNGIMR and IHI in Tanzania. The five subsamples per net were given unique codes as A, B, C, D, and E, were wrapped individually in aluminium foil and stored in a temperature-controlled refrigerator at 4 °C.

Subsamples were received in IHI in December 2020 from PNGIMR, and immediately packed in new aluminium foil and stored in a temperature-controlled refrigerator at 4 °C. The project investigators and facility technicians were blinded to the identity of the products until the end of the study. After all experiments were completed and data were entered, data from PNGIMR cone bioassays was sent to IHI and the blinding was disclosed to the IHI investigators to match the results for each net sample and enable analysis.

## Mosquito rearing and physiological status

### Tanzania

Nulliparous female pyrethroid susceptible *Anopheles gambiae *sensu stricto (*s.s*.) (Ifakara strain) were used; sugar fed, aged between 3–5 days old in cone bioassays, and sugar starved for 6–8 h, aged between 5–8 days old in WHO tunnel tests. The mosquito colony is maintained according to MR4 guidelines [39] at 27 ± 2 °C and a relative humidity of 40%—100%, with ambient (approximately 12 h:12 h) light: dark cycle. Larvae are fed Tetramin fish flakes, and adults are provided with 10% sucrose solution ad libitum and cow blood for egg laying.

### PNG

Nulliparous female pyrethroid susceptible *Anopheles farauti s.s.* were used; sugar fed, aged between 2–5 days old in cone bioassays. The colony is maintained at 28 ± 4 °C and 68 ± 25% relative humidity, with approximately 11 h dark and 12 h light cycle, including a 30 min dusk and 30 min dawn period. The larvae are fed ground fish flakes (Marine Master Tropical Fish Flakes, Australia). The adults are provided 10% sucrose solution ad libitum and human blood for egg laying.

## Cone bioassay procedures

**Fig. 2:**
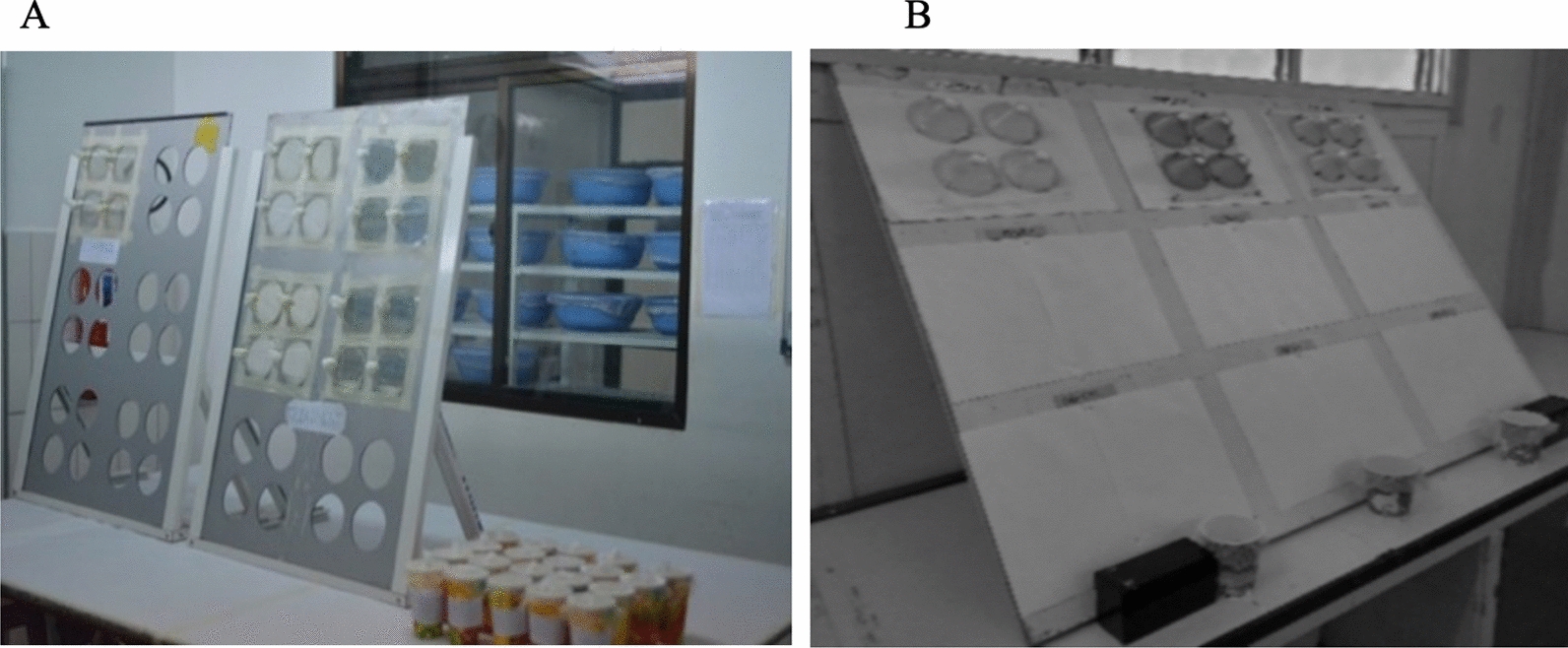
WHO cones fixed on plastic cone board held at 60° in IHI (**A**) and at 45° in PNGIMR (**B**)

On each 25 cm by 25 cm net piece, four standard WHO cones were fixed on a plastic cone board with holes cut and held at 60° [40] in IHI, Tanzania (Fig. 2A) to maximize space and mosquito contact with the ITN, and on a board at 45º [8] in PNGIMR (Fig. 2B). Net pieces were taken from the fridge and kept at room temperature for 2 h before testing. Five laboratory-reared susceptible mosquitoes were placed in each cone for 3-min after which, mosquitoes were removed gently from the cones using a mouth aspirator and kept in individually labelled paper cups, one for each cone. During the holding period, mosquitoes were provided with cotton wool moistened with 10% sucrose solution. Four replicates of 5 mosquitoes were performed on each of the five net pieces making a total of 100 mosquitoes exposed per net. Endpoints measured were KD60 and M24. Mosquitoes exposed to untreated net pieces (negative controls) were tested alongside every replicate to monitor the quality of the bioassay. The bioassays and holding period were carried out at 27 ± 1 °C and at 55%—82% relative humidity in Tanzania and 28 ± 4 °C and at 53%—71% relative humidity in PNG. If the M24 exceeded 10% in a negative control, the test was repeated and (Additional file 2) if the mortality in a negative control was equal or below 10%, the results were adjusted using ‘Abbott’s formula’ [8].

## Tunnel test procedures

WHO tunnel tests were only performed in IHI Tanzania because tunnel tests are not currently established at PNGIMR (Table 1). Two out of five subsamples of nets that did not meet the WHO cone bioassay efficacy criteria, were selected for the WHO tunnel test against susceptible *An. gambiae s.s.* as per WHO guidelines, these were the subsamples that gave mortality closest to the average mortality in the cone bioassay. Tunnel tests were conducted following WHO guidelines [8]. Non-blood fed nulliparous females 5–8 days old, sugar starved for 6–8 h were released in a tunnel made of glass, 60 cm length. At each end of the tunnel, a 25-cm square mosquito cage covered with polyester netting was fitted. At one third of the length, a 25 cm × 25 cm swatch of netting sample was affixed. The surface of netting “available” to mosquitoes is 400 cm^2^ (20 cm × 20 cm), with 9 × 1 cm in diameter holes: one hole is located at the centre of the square; the other eight are equidistant and located at 5 cm from the border. In the shorter section of the tunnel, a small rabbit shaved on its back and restrained in a mesh tunnel was placed as bait. Each rabbit was rested for more than 3 days after use as a bait to ensure welfare. In the cage at the end of the longer section of the tunnel, 100 female mosquitoes were introduced at 21:00 h. The following morning at 09:00 h, the mosquitoes were removed using a mouth aspirator and counted separately from each section of the tunnel, and mortality and blood feeding rates were recorded. The mosquitoes were placed in paper cups and provided with cotton wool moistened with 10% sugar solution. M24 was recorded at around 09:00 h the following day. Mosquitoes exposed to untreated net pieces were used as controls to monitor the quality of the bioassay. The bioassays and holding period were carried out at 27ºC ± 2 °C and 60%—100% relative humidity. Overall mortality was measured by pooling the mortalities of mosquitoes from the two sections of the tunnel. Acceptable feeding success and M24 in controls were > 50% and < 10%, respectively. Any tests that did not achieve the specified control cut off were repeated, all results were adjusted for control mortality using Abbott’s formula [8].

## Sample size

The sample size of four nets per tested product was based on WHO guidelines [8] for testing ITNs. Post hoc power analysis of Cohen’s kappa indicated there was 90% statistical power to detect a difference of up to 20% between facilities [41].

## Statistical analyses

Paper data collection sheets were used to record data, which were double-entered in Microsoft Excel^®^. Data were analysed using Stata^®^ statistical package version 14 (Stata Statistical Software: Release 14. College Station, TX: StataCorp). Proportional KD60 and M24, or BFI and M24 were presented as arithmetic means with their respective 95% confidence intervals (CI). Pass or fail for each net was calculated based on WHO standard efficacy criteria i.e. ≥ 95% KD60 and/or ≥ 80% M24 for cone assay; ≥ 90% BFI and/or ≥ 80% M24 for WHO tunnel test. However, we also considered that the 80% M24 and 95% KD60 thresholds in WHO cone bioassays are subject to stochastic variation. If tests are done using 100 mosquitoes per net as per WHO guidelines, we expect an assay-inherent 95% CI of 71% and 87% around the 80% mortality threshold and a 95% CI of 89% and 98% around the 95% KD60 threshold. ITNs with a mean below the bioefficacy threshold but with 95% CIs that exceeded the bioefficacy threshold of 95% KD60 or 80% M24 were also categorized as pass.

Linear regression was used to analyse the relationship between KD60 and M24. The Spearman rank correlation coefficient (r_s_) was calculated to estimate the degree of correlation between IHI and PNGIMR cone bioassay results for KD60 and M24. Bland–Altman methods [42] were used to assess the agreement between individual measurements of KD60 and M24 from IHI and PNGIMR testing facilities. Cohen’s kappa (κ) was used to assess the degree of agreement between facilities to predict if nets passed or failed WHO cone bioassay threshold criteria.

## Literature review on use of WHO cone bioassays for unused pyrethroid ITN testing

As the current study comprises a limited number of nets it was deemed necessary to conduct a review of literature on cone bioassay for bioefficacy evaluation of unused pyrethroid nets. The aim of the literature review was to investigate how frequently WHO cone bioassays are used to test new, unwashed pyrethroid ITNs and whether cone bioassays are considered a suitable method for this purpose. A search of the literature on ITN efficacy studies, durability studies or WHOPES specification reports published between 2001 and 2021 was conducted in October, 2021 in PubMed and PubMed Central using the keywords “bio-efficacy” or “cone bioassay tests” and “tunnel tests” or “Insecticide treated nets” and “long lasting insecticidal nets” and Google Scholar using the keyword “WHOPES working group meeting”.

**Table 1:** Summary of experiments conducted at Ifakara Health Institute (IHI) and Papua New Guinea Institute of Medical Research (PNGIMR)

Experiment	Bioassay test in IHI	Cone bioassay test in PNGIMR
Number of ITNs tested	20 nets (100 net pieces)	20 nets (100 net pieces)
Mosquitoes exposed	20 per net piece (cone bioassay) 100 per net piece (tunnel tests)	20 per net piece (cone bioassay)
Experiment conditions	27 ± 1 °C (cone bioassay) 55–82% RH (cone bioassay)	28 ± 4 °C (cone bioassay) 53–71% RH (cone bioassay)
	27ºC ± 2 ºC (tunnel tests) 60–100% RH (tunnel tests)	
Mosquito species	Pyrethroid susceptible^*^ *An. gambiae* s.s	Pyrethroid susceptible^*^ *An. farauti* s.s
Mosquito age	3–5 days (cone bioassay) 5–8 days (tunnel tests)	2–5 days (cone bioassay)
WHO efficacy criteria	≥ 95%KD60 or ≥ 80% M24 (cone bioassay)	≥ 95% KD60 or ≥ 80% M24 (cone bioassay)
	≥ 90% BFI and/or ≥ 80% M24 (tunnel tests)	

*RH* relative humidity, *KD60* knockdown measured at 60 min (sublethal incapacitation), *M24* mortality measured at 24 h post exposure, *BFI* blood feeding inhibition

^*^Sugar fed *Anopheles gambiae* s.s (Ifakara) *and Anopheles farauti* s.s were confirmed to be 100% susceptible to alpha-cypermethrin, deltamethrin and permethrin insecticides at 1× WHO discriminating concentration at the time of evaluation

Overall, the literature search identified 2,362 titles (PubMed: 87 titles, PubMed Central: 1,604 titles and Google Scholar: 671 titles). Titles were further screened for reports using standard WHO evaluation methods on unused pyrethroid ITNs with *Anopheles* mosquitoes that reported both KD60 and M24. This resulted in seventy publications being fully screened and sixty being included in the final selection. Data extracted from selected publications included ITN type (brand name, active ingredient, manufacturing technology, manufacturing date or year, batch/lot number), bioassay results (mainly KD60 and M24), the *Anopheles* strain used in the bioassays and where and when the study was conducted.

## Results

### Bioefficacy of unused ITNs against susceptible *An. gambiae* in IHI Tanzania and susceptible *An. farauti* in PNGIMR

At IHI, Tanzania, 13/20 nets (65%) met the WHO cone bioassay bioefficacy criteria of ≥ 95% KD60 and/or ≥ 80% M24. The seven nets that did not meet cone bioassay criteria, met bioefficacy criteria of ≥ 90% BFI and ≥ 80% M24 in the WHO tunnel tests. At PNGIMR, 8/20 nets (40%) met WHO cone bioassay bioefficacy criteria (Table 2).

**Table 2:** WHO cone bioassay and tunnel test results on tested unused pyrethroid ITNs in IHI Tanzania (with susceptible *Anopheles gambiae* s.s.) and PNGIMR (with susceptible *Anopheles farauti* s.s.) testing facilities

Test Item	Cone test		#Nets pass in cone	Tunnel test*		#Nets pass combined cone and tunnel tests
	% KD60 (95% Cl)	%24-h Mortality (95% Cl)		% Feeding inhibition (95% Cl)	%24-h Mortality (95% Cl)	
IHI						
PermaNet^®+^	100	99.7 (99.2–100)	4/4			4/4
PermaNet^®++^	80.0 (76.0–84.0)	22.3 (17.8–26.7)	1/4	98.3 (94.7–100)	97.8 (94.5–100)	4/4
Interceptor^®^	85.8 (82.6–88.9)	37.9 (32.0–43.7)	1/4	99.7 (99–100)	99.5 (98.5–100)	4/4
SafeNet^®^	97.3 (95.6–98.9)	61.1 (55.2–67.0)	3/4	100	100	4/4
Yorkool^®^	96.8 (94.8–98.7)	59.7 (56.1–63.3)	4/4			4/4
PNGIMR						
PermaNet^®+^	96.4 (92.3–100)	99.6 (98.8–100)	4/4			
PermaNet^®++^	37.1 (29.3–44.9)	25.9 (14.1–37.6)	0/4			
Interceptor^®^	79.3 (72.7–85.8)	72.8 (66.7–78.8)	0/4			
SafeNet^®^	82.0 (75.1–88.9)	81.0 (74.8–87.2)	1/4			
Yorkool^®^	87.3 (83.5–91.0)	88.5 (83.9–93.1)	3/4			

^*^Tunnel test performed to the nets that did not meet optimal efficacy criteria (≥ *95% KD60 and/or* ≥ *80% M24) in cone bioassay at IHI,* ^+^ *PermaNet*^*®*^* 2.0 manufactured in 2012,* ^++^*PermaNet*^*®*^* 2.0 manufactured in 2019, # Number of*

### The relationship between 60-min knockdown and 24-h mortality in cone bioassay

In IHI, the relationship between knockdown and mortality measured by regression was 0.36 (95% CI: 0.15–0.57 *p* = 0.002) indicating that KD60 and M24 were not closely related. While in PNGIMR the relationship was 0.78 (95% CI: 0.64–0.93 *p* < 0.001) indicating that there was high probability of knocked down mosquitoes dying.

### Level of correlation between IHI and PNGIMR on KD60 and M24 in WHO cone bioassays

**Fig. 3:**
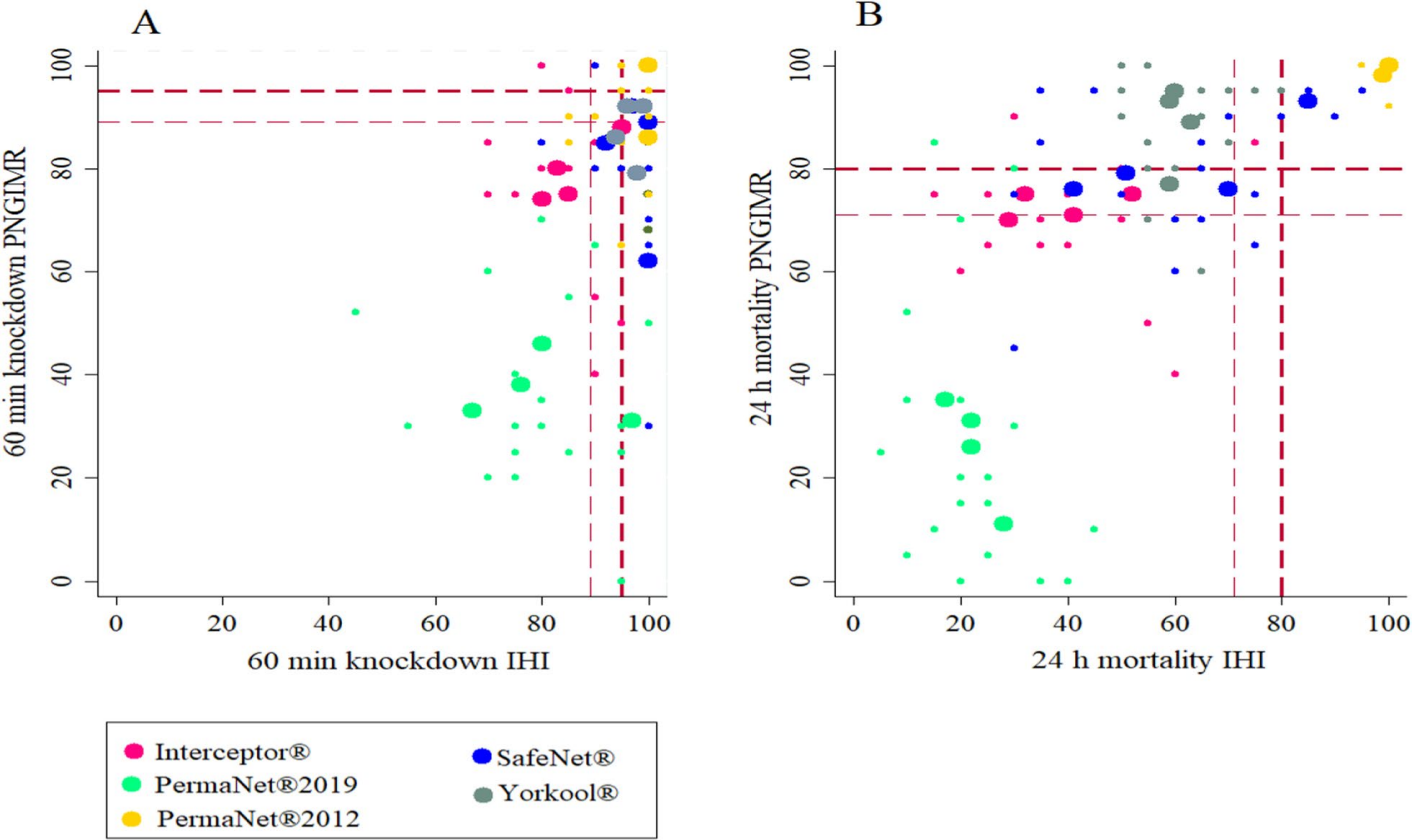
Correlation of cone bioassay tests results between IHI and PNGIMR testing facilities. Thick dashed lines are the WHO threshold 95% KD60 (**A**) and 80% M24 (**B**). Thin dashed lines indicate these assay-inherent 95% (lower) CIs of these thresholds. Large dots represent averages per sampled nets (4 per net type) and small dots represent all subsamples (5 per net)

Correlation between IHI and PNGIMR results was statistically highly significant but with a stronger association between M24 results (r = 0.9, *p* < 0.0001, n = 20) than between KD60 results (r = 0.6, *p* = 0.002, n = 20) (Fig. 3). While there was some discrepancy in results on KD60 and M24, the bioassay was predictive of pass or failure. Those nets that failed WHO bioefficacy criteria in IHI also failed in PNGIMR except for two nets (5 and 12). PermaNet^®^2012 ITNs exceeded thresholds of KD60 or M24 in both facilities i.e., those nets passed WHO bioefficacy criteria (KD60 or M24) in IHI also passed in PNGIMR with the exception of net 2 (Fig. 3). Overall results show a higher knockdown rate and lower mortality rate at IHI relative to PNGIMR.

### Agreement of cone bioassay at IHI and PNGIMR testing facilities based on KD60 and M24

**Fig. 4:**
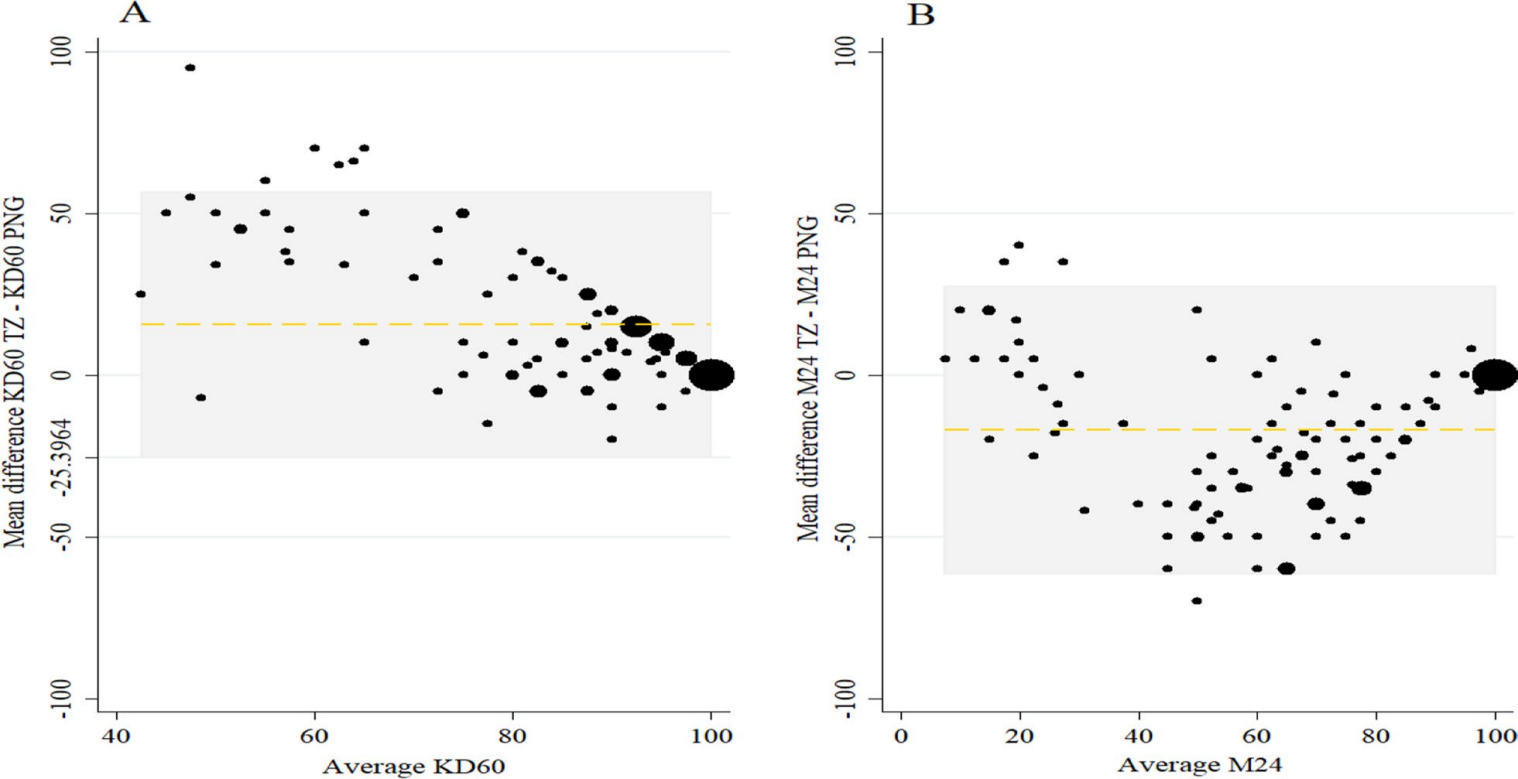
Bland-Altman Plot showing the mean difference (y axis) plotted against the average value from both sites (x) **A** KD60 and **B** M24. For KD60 Mean difference (limits of agreement) was 15.5 (−25.4–56.5) and for M24 Mean difference (limits of agreement) was −17.0 (−61.4–27.3). At lower mean values of knockdown, the agreement between the two testing facilities was lower than at higher mean values of knockdown but there was a consistent difference in mean difference in M24 measured at each testing facility

Using Bland–Altman Plot a more consistent agreement in M24 results than for KD60 was observed (Fig. 4). The limits of agreement for both endpoints were wide: KD60 mean difference (limits of agreement) 15.5 (− 25.4 to 56.5) and M24 − 17.0 (− 61.4 to 27.3). As expected, agreement was highest among the most efficacious nets with high KD60 and M24.

### Agreement of cone bioassay at IHI and PNGIMR testing facilities based on WHO pass/fail criteria

**Fig. 5:**
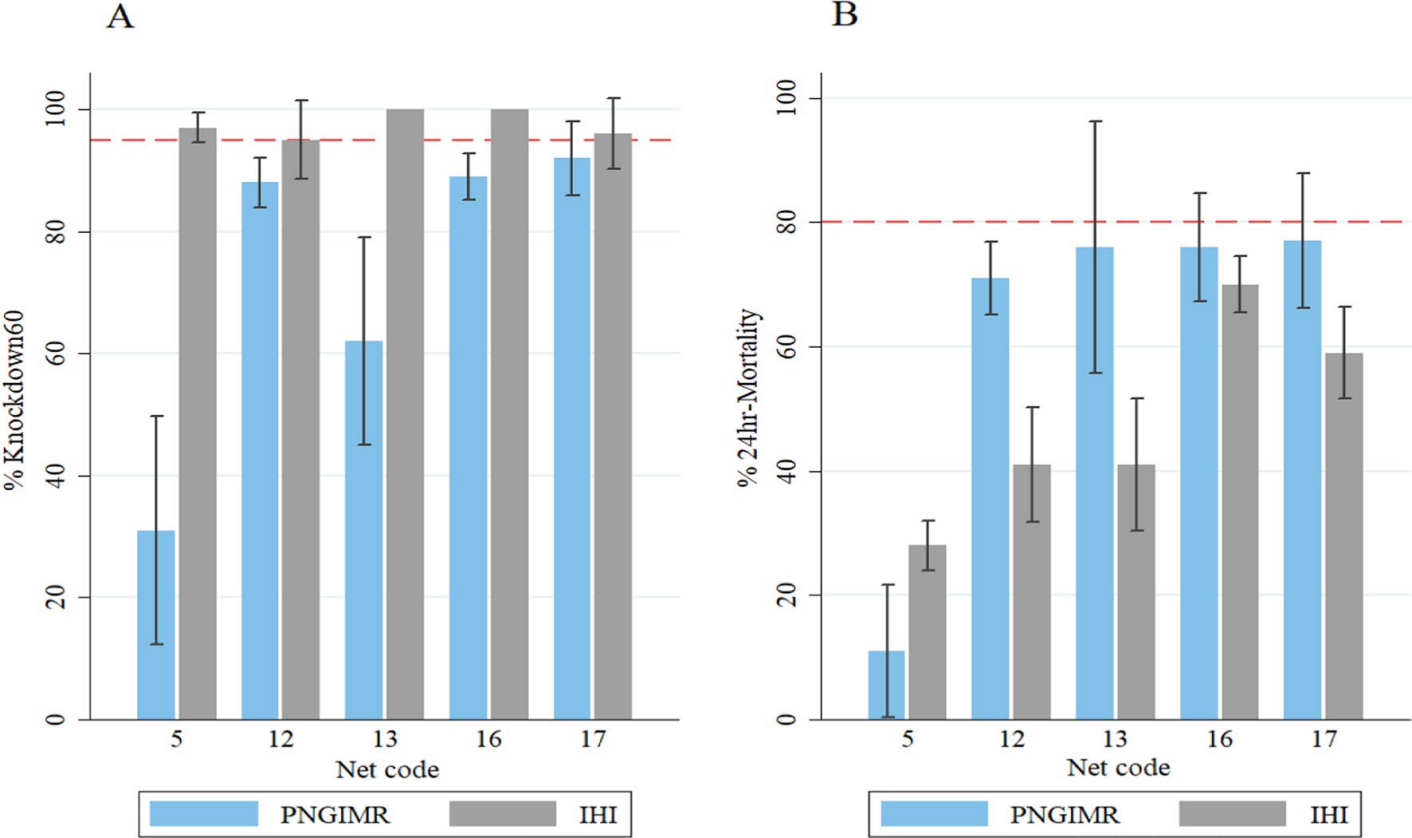
Bioefficacy of the five ITNs that demonstrated discordant results of pass or fail between facilities. Each ITN passed efficacy criteria in IHI using the bioefficacy criterion of 95% KD60 **A** but did not reach the optimal bioefficacy criterion of 80% 24 h mortality **B**. Three of the nets showed mean 24 h mortality close to 80% at PNGIMR with confidence intervals that overlapped the optimal bioefficacy threshold of 80% mortality **B**. Dashed lines are the WHO thresholds for 95% KD60 and 80% M24

To account for variability inherent to the cone bioassay, the mortality pass rate was set at 71% and the KD60 rate to 89% (i.e., the lower 95% CIs of each). Thereafter, IHI and PNGIMR data agreed for n = 18 (90%) of the ITNs (based on combined estimate from 5 net pieces), classifying n = 6 (30%) as “fail” at both facilities and n = 12 (60%) as “pass” at both facilities (Table 3). Also of note, of the five nets that demonstrated discordant pass or fail between facilities using the standard WHO bioefficacy thresholds (ignoring variability), three ITNs were re-categorized as pass in PNGIMR using the revised threshold. These nets had passed on KD60 at IHI and although failed both bioefficacy criteria at PNGIMR but their 95% confidence interval overlapped the optimal bioefficacy threshold of 80% M24 (Fig. 5).

**Table 3:** Contingency analysis for cone bioassays conducted in IHI and PNGIMR to classify the n = 20 ITNs (mean value of 5 net pieces from each ITN) into ‘pass’ and ‘fail’ categories based on mean values for M24 and KD60, using the WHO bioefficacy criteria and the inherent lower CI of the per-protocol cone bioassay as threshold for pass or fail

		PNGIMR		Total N (%)
		Pass N (%)	Fail N (%)	
IHI	Pass	12^a^ (100)	2^c^ (25)	14 (70)
	Fail	0^b^ (0)	6^d^ (75)	6 (30)
	Total	12 (60)	8 (40)	20 (100)

^‘a’^ and ‘^d’^ the number of nets agreed results at both testing facilities, ‘^b’^ and ‘.^c’^ the number of nets with discrepant results between testing facilities

**Fig. 6:**
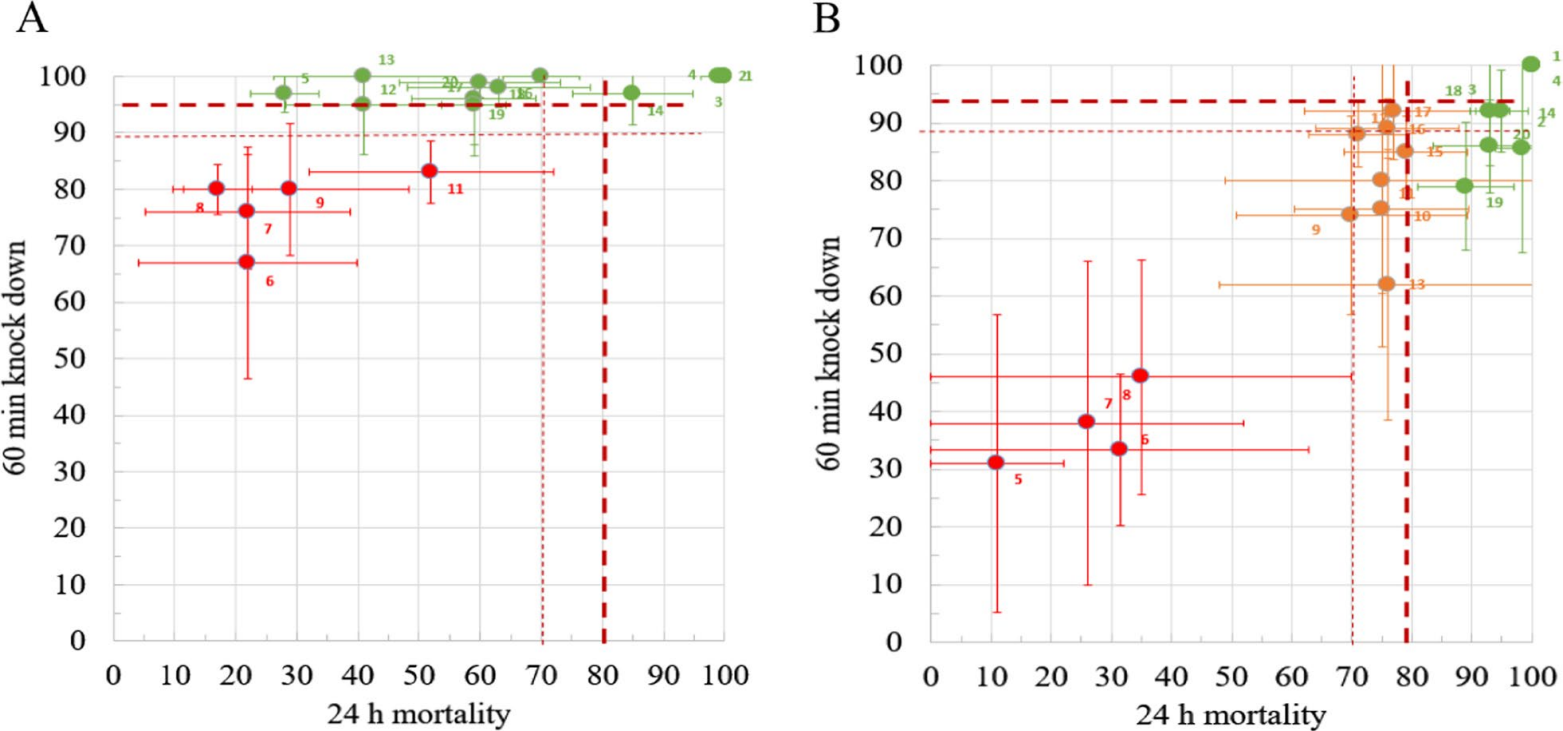
Correlation between M24 and KD60 at IHI **A** and PNGIMR **B**. ITNs passing (green) or failing (red) based on stringent cut-off WHO cone bioassay criteria of 80% M24 and 95% KD60. ‘Borderline’ nets for which the mean KD or M24 values are within the margin of stochastic error (95% CI) inherent to WHO cone bioassays based on the total number of mosquitoes used (n = 100) are shown in amber. Thick dashed lines are the WHO thresholds 95% KD60 and 80% M24. Thin dashed lines indicate these assay-inherent 95% (lower) CIs of these thresholds

The agreement between the bioefficacy results at IHI and PNGMR to predict ITN pass or fail was good with κ = 0.79 (0.53–1.00) and 90% accuracy. The two discrepant nets (net 5 and net 12) passed at IHI on KD60 but not M24 (Fig. 5). No nets with M24 exceeding 80% failed at either facility, while the majority of nets that passed at IHI, passed only on KD60 (Fig. 6).

### Literature review on utility of cone bioassay for pyrethroid ITNs testing

**Fig. 7:**
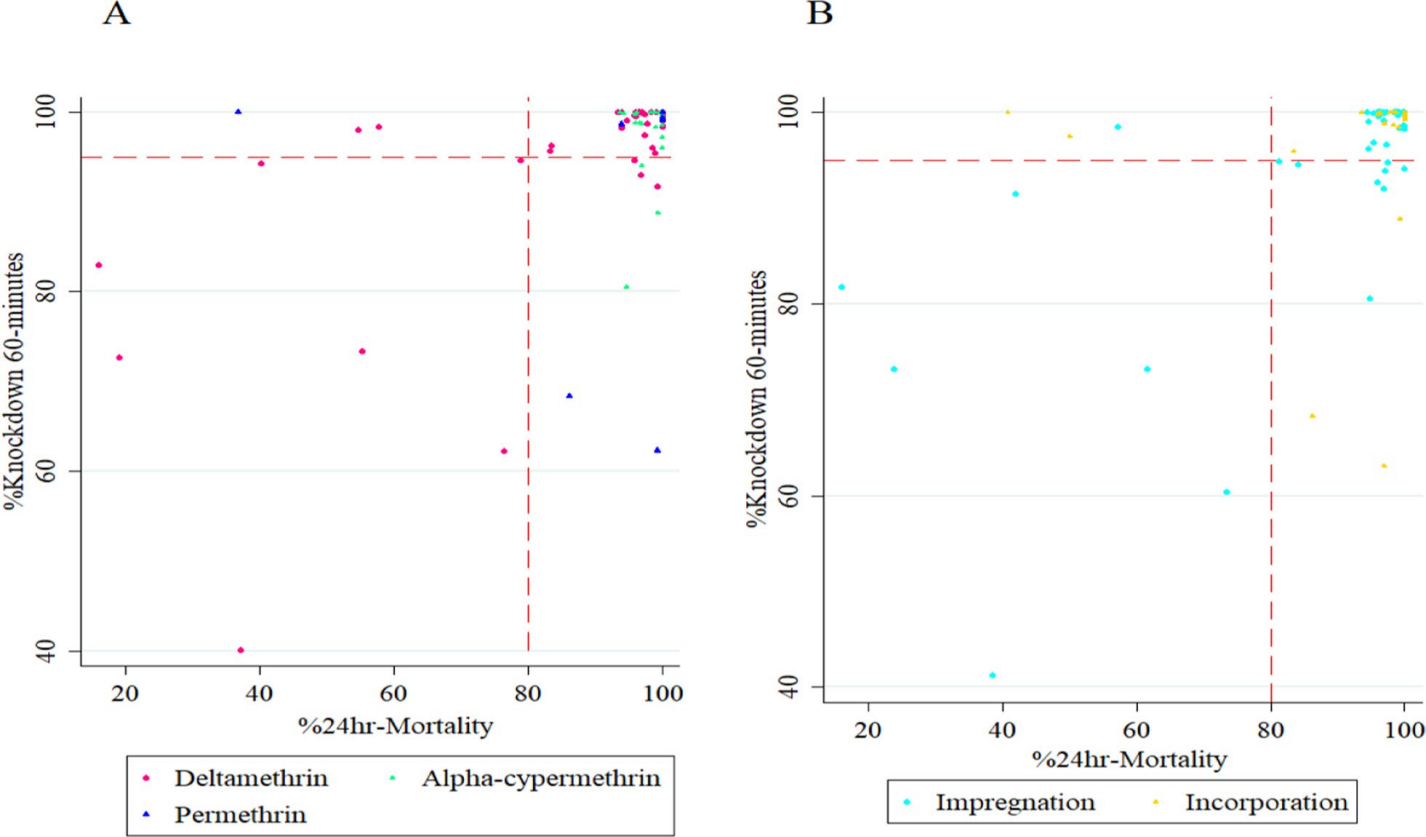
Results from the literature review **A** Relationship between KD60 and M24 in WHO cone bioassays with pyrethroid ITNs (deltamethrin, alpha-cypermethrin and permethrin) using *Anopheles* mosquitoes. **B** Relationship between KD60 and M24 in ITNs grouped by production technology. Dashed lines are the WHO threshold 95% KD60 and 80% M24

The literature review on the use of WHO cone bioassays for pyrethroid ITN testing showed that the vast majority of unused pyrethroid ITNs scored high KD60 and M24 (Fig.7). On average KD60 was 96% (95% CI: 94–98) and M24 was 92% (95% CI: 88–96). From the 83 observations with unwashed ITNs that included both KD60 and M24 observations, mainly with *An. gambiae* s.s (63/83) and mainly with deltamethrin coated ITNs (51/83) only 12 reported KD60 below 95% (Table 4). Interestingly, even permethrin ITNs gave very high knockdown 89% (95% CI: 74–100) and mortality 89% (95% CI: 68–100) in studies published between 2008 and 2017.

**Table 4:** The literature review on cone bioassays for pyrethroid ITN testing

Author/Report	Location	Pyrethroid ITN^*^	Active Ingredients	Production technology	Year^#^	Mosquito strains (Susceptible)	KD60	M24
Abilio and colleagues, 2015 [22]	Mozambique	Interceptor^®^	Alpha-cypermethrin	Impregnation		*An. arabiensis*	80.56	98.84
Abilio and colleagues, 2015 [22]	Mozambique	PermaNet^®^ 2.0	Deltamethrin	Impregnation		*An. arabiensis*	94.72	100
Abilio and colleagues, 2015 [22]	Mozambique	Olyset Net^®^	Permethrin	Incorporation		*An. arabiensis*	68.33	90.36
Agossa and colleagues, 2014 [84]	Benin	PermaNet^®^ 2.0	Deltamethrin	Impregnation		*An. gambiae* (Kisumu strain)	100	100
Ahogni and colleagues, 2019 [43]	Benin	Yorkool^®^	Deltamethrin	Impregnation	2017	*An. gambiae* (Kisumu strain)	62	74
Allossogbe and colleagues, 2017 [85]	Benin	PermaNet^®^ 2.0	Deltamethrin	Impregnation	2015–2016	*An. gambiae* (Kisumu strain)	93.33	100
Allossogbe and colleagues, 2017 [85]	Benin	Olyset Net^®^	Permethrin	Incorporation	2015–2016	*An. gambiae* (Kisumu strain)	100	100
Bagheri and colleagues, 2017 [44]	Iran	PermaNet^®^ 2.0	Deltamethrin	Impregnation	2016	*An. stephensi*	74	22
Bhatt and colleagues, 2012 [24]	India	Interceptor^®^	Alpha-cypermethrin	Impregnation	2006	*An. culicifacies*	96.7	100
Camara and colleagues, 2018 [80]	Côte d’Ivoire	Interceptor^®^	Alpha-cypermethrin	Impregnation		*An. gambiae* (Kisumu strain)	97	99
Castellanos and colleagues, 2021 [19]	Guatemala	PermaNet^®^ 2.0	Deltamethrin	Impregnation	2012	*An. albimanus*	100	100
Clegban and colleagues, 2021 [86]	Côte d’Ivoire	Yahe^®^	Deltamethrin	Impregnation	2014	*An. gambiae* (Kisumu strain)	100	97.2
Clegban and colleagues, 2021 [86]	Côte d’Ivoire	PandaNet^®^ 2.0	Deltamethrin	Incorporation	2014	*An. gambiae* (Kisumu strain)	100	100
Graham and colleagues, 2005 [18]	Iran	PermaNet^®^ 2.0	Deltamethrin	Impregnation	2000	*An. stephensi* (Beech strain)	100	97.7
Kilian and colleagues, 2008 [48]	Montpellier, France	PermaNet^®^ 1.0	Deltamethrin	Impregnation	2000	*An. gambiae* (Kisumu strain)	95	80
Kilian and colleagues, 2008 [48]	CDC Atlanta, USA	PermaNet^®^ 2.0	Deltamethrin	Impregnation	2002	*An. gambiae* (Kisumu strain)	95	80
Kweka and colleagues, 2011 [87]	Tanzania	PermaNet^®^ 2.0	Deltamethrin	Impregnation	2005	*An. gambiae* (Kisumu strain)	100	100
Kweka and colleagues, 2017 [78]	Tanzania	PermaNet^®^ 2,0	Deltamethrin	Impregnation		*An. gambiae* (Kisumu strain)	100	100
Kweka and colleagues, 2019 [88]	Tanzania	MagNet^®^	Alpha-cypermethrin	Incorporation		*An. gambiae* (Kisumu strain)	90.4	100
Kweka and colleagues, 2019 [88]	Tanzania	DuraNet^®^	Alpha-cypermethrin	Incorporation		*An. gambiae* (Kisumu strain)	100	100
Mahande and colleagues, 2018 [89]	Tanzania	DuraNet^®^	Alpha-cypermethrin	Incorporation	2015	*An. gambiae* (Kisumu strain)	100	100
Malima and colleagues, 2013 [23]	Tanzania	Interceptor^®^	Alpha-cypermethrin	Impregnation		*An. gambiae s.l*	100	100
Mussa and colleagues, 2020 [90]	Tanzania	DawaPlus^®^	Deltamethrin	Impregnation	2019	*An. gambiae*	100	92.5
Ngufor and colleagues, 2020 [91]	Benin	Royal Sentry^®^	Alpha-cypermethrin	Incorporation		*An. gambiae *(Kisumu strain)	100	98
Okia and colleagues, 2013 [17]	Uganda	PermaNet^®^ 2.0	Deltamethrin	Impregnation	Started 2011	*An. gambiae* (Kisumu strain)	100	100
Okia and colleagues, 2013 [17]	Uganda	Interceptor^®^	Alpha-cypermethrin	Impregnation	Started 2011	*An. gambiae* (Kisumu strain)	95	100
Okia and colleagues, 2013 [17]	Uganda	Olyset Net^®^	Permethrin	Incorporation	started 2011	*An. gambiae* (Kisumu strain)	100	100
Pennetier and colleagues, 2013 [92]	Malanville, Benin	Olyset Net^®^	Permethrin	Incorporation		*An. gambiae* (Kisumu strain)	64	100
Rafinejad and colleagues, 2008 [21]	Iran	PermaNet^®^	Deltamethrin	Impregnation		*An. stephensi*	100	94.9
Rafinejad and colleagues, 2008 [21]	Iran	Olyset Net^®^	Permethrin	Incorporation		*An. stephensi*	100	97
Randriamaherijaona and colleagues, 2017 [45]	Madagascar	Royal Sentry^®^	Alpha-cypermethrin	Incorporation		*An. arabiensis*	100	90.2
Sood and colleagues, 2011 [93]	India	PermaNet^®^ 2.0	Deltamethrin	Impregnation		*An. stephensi*	100	100
Sood and colleagues, 2011 [93]	India	Olyset Net^®^	Permethrin	Incorporation		*An. stephensi*	100	100
Vinit and colleagues, 2020 [46]	Papua New Guinea	PermaNet^®^ 2.0	Deltamethrin	Impregnation	Between 2007 and 2012	*An. farauti*	96.48	98.72
Vinit and colleagues, 2020 [46]	Papua New Guinea	PermaNet^®^ 2.0	Deltamethrin	Impregnation	Between 2013 and 2019	*An. farauti*	41.23	40.12
WHO, 2004 [94]	Montipellier, France	PermaNet^®^ 1.0	Deltamethrin	Impregnation	2–4 December, 2003	*An. gambiae* (Kisumu strain)	100	100
WHO, 2004 [94]	Benin	PermaNet^®^ 2.0	Deltamethrin	Impregnation	2–4 December, 2003	*An. gambiae* (Kisumu strain)	100	100
WHO, 2004 [94]	Montipellier, France	PermaNet^®^ 1.0	Deltamethrin	Impregnation	2–4 December, 2003	*Cx. quinquefasciatus*	100	100
WHO, 2007 [35]	Malanville, Benin	Interceptor^®^	Alpha-cypermethrin	Impregnation	11–14 December, 2006	*An. gambiae*	100	100
WHO, 2007 [35]	Montipellier, France	Hiking Group^®^	Deltamethrin	Impregnation	11–14 December, 2006	*An. gambiae* (Kisumu)	100	95
WHO, 2007 [35]	Montipellier, France	Yorkool^®^	Deltamethrin	Impregnation	11–14 December, 2006	*An. gambiae* (Kisumu)	83	16
WHO, 2007 [35]	Montipellier, France	Netto Group^®^	Deltamethrin	Impregnation	11–14 December, 2006	*An. gambiae* (Kisumu)	95	100
WHO, 2007 [35]	Montipellier, France	PermaNet^®^ 2.0	Deltamethrin	Impregnation	11–14 December, 2006	*An. gambiae* (Kisumu)	100	100
WHO, 2008 [13]	Kyenjonjo, Uganda	PermaNet^®^ 2.0	Deltamethrin	Impregnation	11–14 December, 2006	*An. gambiae* (Kisumu)	95	95
WHO, 2008 [13]	Montipellier, France	Dawaplus^®^	Deltamethrin	Impregnation	10–13 December, 2007	*An. gambiae* (Kisumu)	93	39
WHO, 2008 [13]	Kou Valley, Bukina Faso	Netprotect^®^	Deltamethrin	Incorporation	10–13 December, 2007	*An. gambiae* (Kisumu)	100	100
WHO, 2008 [13]	Kou Valley, Bukina Faso	DuraNet^®^	Alpha-cypermethrin	Incorporation	10–13 December, 2007	*An. gambiae* (Kisumu)	100	100
WHO, 2008 [13]	WHOPES supervised studies	DuraNet^®^	Alpha-cypermethrin	Incorporation	10–13 December, 2007	*An. gambiae* (Kisumu)	100	98
WHO, 2008 [13]	Muheza, Tanzania	DuraNet^®^	Alpha-cypermethrin	Incorporation	10–13 December, 2007	*An. gambiae* (Kisumu)	100	100
WHO, 2009 [95]	Melanville, North Benin	PermaNet^®^ 2.5	Deltamethrin	Impregnation	8–11 December, 2008	*An. gambiae* (Kisumu)	100	100
WHO, 2009 [95]	Melanville, North Benin	PermaNet^®^ 2.0	Deltamethrin	Impregnation	8–11 December, 2008	*An. gambiae* (Kisumu)	100	100
WHO, 2009 [95]	Kilimanjaro district, Tanzania	PermaNet^®^ 2.5	Deltamethrin	Impregnation	8–11 December, 2008	*An. gambiae* (Kisumu)	100	100
WHO, 2009 [95]	Kilimanjaro district, Tanzania	PermaNet^®^ 2.0	Deltamethrin	Impregnation	8–11 December, 2008	*An. gambiae* (Kisumu)	100	100
WHO, 2010 [16]	Montipellier, France	Yorkool^®^	Deltamethrin	Impregnation	28–30 July, 2009	*An. gambiae* (Kisumu)	100	55
WHO, 2010 [16]	Montipellier, France	PermaNet^®^ 2.0	Deltamethrin	Impregnation	28–30 July, 2009	*An. gambiae* (Kisumu)	100	100
WHO, 2010 [16]	Malanville, Benin	DawaPlus^®^	Deltamethrin	Impregnation	28–30 July, 2009	*An. gambiae* (Kisumu)	100	100
WHO, 2010 [16]	Malanville, Benin	DawaPlus^®^ 2.0	Deltamethrin	Impregnation	28–30 July, 2009	*An. gambiae* (Kisumu)	100	100
WHO, 2010 [16]	Muheza, Tanzania	DawaPlus^®^ 2.0	Deltamethrin	Impregnation	28–30 July, 2009	*An. gambiae* (Kisumu)	100	100
WHO, 2011 [96]	Montipellier, France	Yahe^®^	Deltamethrin	Impregnation	11–15 April, 2011	*An. gambiae* (Kisumu)	100	100
WHO, 2011 [96]	Montipellier, France	PermaNet^®^ 2.0	Deltamethrin	Impregnation	11–15 April, 2011	*An. gambiae* (Kisumu)	100	100
WHO, 2011 [96]	Montipellier, France	Royal Sentry^®^	Alpha-cypermethrin	Incorporation	11–15 April, 2011	*An. gambiae* (Kisumu)	100	100
WHO, 2011 [96]	Montipellier, France	DuraNet^®^	Alpha-cypermethrin	Incorporation	11–15 April, 2011	*An. gambiae* (Kisumu)	100	100
WHO, 2011 [96]	Montipellier, France	MagNet^®^	Alpha-cypermethrin	Incorporation	11–15 April, 2011	*An. gambiae* (Kisumu)	100	100
WHO, 2011 [96]		LifeNet^®^	Deltamethrin	Incorporation	11–15 April, 2011	*An. gambiae* (Kisumu)	100	100
WHO, 2012 [97]	India	Interceptor^®^	Alpha-cypermethrin	Impregnation	18–22 June, 2012	*An. culicifacies*	97.8	98
WHO, 2012 [97]	Muheza, Tanzania	Interceptor^®^	Alpha-cypermethrin	Impregnation	18–22 June, 2012	*An. gambiae* (Kisumu)	100	99
WHO, 2012 [97]	Benin	Olyset Net^®^	Permethrin	Incorporation	18–22 June, 2012	*An. gambiae* (Kisumu)	100	37
WHO, 2013 [98]	Mae Sot District, Thailand	PermaNet^®^ 2.0	Deltamethrin	Impregnation	22–30 July, 2013	*An. culicifacies*	100	100
WHO, 2013 [98]	Muheza, Tanzania	Yahe^®^	Deltamethrin	Impregnation	22–30 July, 2013	*An. gambiae* (Kisumu)	100	100
WHO, 2013 [98]	Mae Sot District, Thailand	Yahe^®^	Deltamethrin	Impregnation	22–30 July, 2013	*An. minimus*	73	58
WHO, 2013 [98]	Muheza, Tanzania	PermaNet^®^ 2.0	Deltamethrin	Impregnation	22–30 July, 2013	*An. gambiae* (Kisumu)	100	100
WHO, 2013 [98]	Rourkela, India	DuraNet^®^	Alpha-cypermethrin	Incorporation	22–30 July, 2013	*An. culicifacies*	100	100
WHO, 2015 [15]	WHOPES supervised studies	Yahe^®^	Deltamethrin	Impregnation	29 June-1 July, 2015	*An. gambiae* (Kisumu)	93	97
WHO, 2015 [15]	Montipellier, France	SafeNet^®^	Alpha-cypermethrin	Impregnation	29 June-1 July, 2015	*An. gambiae* (Kisumu)	100	99.5
WHO, 2015 [15]	Montipellier, France	SafeNet^®^	Alpha-cypermethrin	Impregnation	29 June-1 July, 2015	*An. gambiae* (Kisumu)	100	100
WHO, 2015 [15]	Montipellier, France	Interceptor^®^	Alpha-cypermethrin	Impregnation	29 June-1 July, 2015	*An. gambiae* (Kisumu)	100	100
WHO, 2015 [15]	Montipellier, France	PermaNet^®^ 2.0	Deltamethrin	Impregnation	29 June-1 July, 2015	*An. gambiae* (Kisumu)	100	100
WHO, 2015 [15]	Côte d’Ivoire	PermaNet^®^ 2.0	Deltamethrin	Impregnation	29 June-1 July, 2015	*An. gambiae* (Kisumu)	100	100
WHO, 2015 [15]	WHOPES supervised studies	MiraNet^®^	Alpha-cypermethrin	Incorporation	29 June– 1 July, 2015	*An. gambiae* (Kisumu)	100	100
WHO, 2015 [15]	Côte d’Ivoire	Panda Net^®^ 2.0	Deltamethrin	Incorporation	29 June– 1 July, 2015	*An. gambiae* (Kisumu)	100	100
WHO, 2015 [15]	WHOPES supervised studies	Panda Net^®^ 2.0	Deltamethrin	Incorporation	29 June– 1 July, 2015	*An. gambiae* (Kisumu)	97	51
WHO, 2019 [99]	Ifakara Health Institute, Tanzania	Royal Sentry^®^ 2.0	Alpha-cypermethrin	Incorporation	2017	*An. gambiae* (Ifakara strain)	100	100
WHO, 2020 [100]	Reference laboratory	Tsara Soft^®^	Deltamethrin	Incorporation	2019	*An. dirus*	95	80

^*^Include new unused, old unused or unwashed Long-Lasting Insecticidal Nets; ^#^Many reviewed studies did not report ITNs manufactured date/year, hence in this review encompass and report either manufactured year/distributed year/tested year or date of meeting

*KD60* knockdown measured at 60 min (sublethal incapacitation), *M24* mortality measured at 24 h post exposure

## Discussion

The present study explored the utility of cone bioassays for pre-delivery QA of pyrethroid ITNs in two test facilities using different *Anopheles* mosquitoes to test the assumption that cone bioassays are consistent and reproducible across locations, mosquito strains, and laboratories, and could be conducted in addition to physiochemical tests currently recommended for QA of ITNs [31]. This study specifically compared the test results for unused pyrethroid ITNs from PNG using susceptible *An. gambiae s.s* and susceptible *An. farauti s.s.* WHO tunnel tests were used as a supplementary test in IHI to confirm bioefficacy of the nets that did not meet bioefficacy thresholds in cone bioassays. Consistency of bioefficacy results on tested ITNs between IHI and PNGIMR was demonstrated (κ = 79 and 90% accuracy), based on pass/fail categories (Table 3), although absolute agreement between IHI and PNGIMR testing facilities was not observed, especially for those nets with low M24.

In this study, after modifying the pass criteria to account for inherent stochastic variation and systematic bias there was good agreement between the facilities indicating that the cone bioassay is a sensitive method to identify those nets with sufficient insecticide doses on the net surface to kill and incapacitate pyrethroid susceptible mosquitoes. It may, therefore, provide a means to identify nets with suboptimal insecticide doses on the net surface even using different *Anopheles* strains in different laboratories. Most previous studies identified from the literature review using cone bioassay tests reported bioefficacy above WHO critical thresholds for unused pyrethroid ITNs [15–26]. However, a handful of studies reported bioefficacy below WHO critical thresholds in Benin [43], Iran [44], Madagascar [45], and PNG [46]. The reasons for this are unclear but our study corroborates the recent findings from PNG [46]. However, it is known that cone bioassay results can be affected by ITN characteristics i.e. manufacturing processes [47–49], and possibly poor shipping or storage conditions [50]; as well as bioassay methods including sample preparation e.g. using a net sample straight from the fridge, mosquito age [51, 52] and fitness [53], test procedures [40], temperature [54–56] and inter-operator variability [31].

As the cone test uses biological systems there are many factors that can affect the results obtained that need to be carefully controlled. These can be grouped into (1) mosquito rearing, (2) infection control, (3) environmental conditions, (4) mosquito related factors and (5) conduct of the cone test.

### Mosquito rearing

It is critical to standardise temperature because larval rearing temperature affects mosquito fitness and may alter their resistance to insecticides [57]. Rearing mosquitoes with an incorrect light dark cycle may decrease mosquito survival [58]. Mosquito larval nutrition affects the size of mosquitoes and, therefore, may also affect their susceptibility to insecticides [52]. Optimal mosquito rearing procedures are outlined in the MR4 Guidelines [39].

### Infection control

Preparation of the testing room and mosquito holding area before the conduct of the cone test is important. The laboratory and holding rooms need to be kept clean in order to prevent mosquito infection with microorganisms that may alter the observed mortality [59]. Mosquito infection with pathogens reduces their host seeking and egg laying [60].

### Environmental conditions

There is some evidence that humidity can also affect mosquito mortality observed after insecticide exposure [61] and it is known to affect mosquito survival [62] and should therefore be carefully maintained during mosquito holding post-exposure. Mosquito detoxification has a periodicity [63] that follows the natural circadian rhythm of the mosquito [64] so it is important to conduct studies at a similar time each day to minimize heterogeneity between observations.

### Mosquitoes

Using mosquitoes with standardised age and nutritional status is essential to allow the comparability of assays between laboratories. The age [65, 66] and nutritional status (blood [65] and sugar [67]) of mosquitoes alters their susceptibility to insecticides. The time that a mosquito received a blood meal relative to contact with an insect growth regulator or juvenile hormone analogue can impact the results of the bioassay [68]. Careful transport of mosquitoes from the insectary to the test room in sealed containers and allowing mosquitoes to acclimate to the test room before bioassay will minimize physiological stress and its effects on metabolic and physiological status and so avoid possible bias in observed mortality. It is important to avoid overuse of the colony so that the colony becomes depleted and individual mosquito fitness is compromised.

### Conduct of cone test

For ITN samples that are refrigerated, allowing the ITNs to return to room temperature before testing is important. This is because pyrethroids have a temperature dependent toxicity [69] and failure to test the ITNs at the correct temperature may introduce bias into the observed mortality. The angle at which the WHO cone bioassay is performed considerably affects the time mosquitoes spend in contact with the net, and subsequently 24 h mortality. It is advised to use the cone test at 45º or 60º angle to maximize mosquito contact with the treated surface of the ITN [40]. Placement of ITN samples on the board should be done without stretching or bunching the ITN material as this affects the amount of treated netting under the cone and consequently the treated surface is available to the mosquito. To enable comparability of results from different test facilities, standard cone (12 cm diameter, available from WHO) should be used to standardize the surface area of netting available to mosquitoes. Cutting a hole in the board and using plastic stoppers so that mosquitoes can only rest on the ITN sample for the exposure time (as done at IHI) helps to minimize heterogeneity in results. For the purposes of comparability between testing facilities and time points, it is critical to evaluate ITNs at a standard temperature of 27 ± 2ºC. Conducting studies at a different temperature can affect the observed results. A bimodal temperature-activity distribution has been reported in several insecticides and mosquito species [54, 55, 70, 71] and 27 ± 2ºC gives a conservative measurement of mortality. Temperature affects the way in which pyrethroids work in insects. Initial symptoms of Type I pyrethroids are positively correlated with temperature, the toxic action (release of neurotransmitter and conduction block) is negatively correlated with temperature [69] whereas other insecticide classes tend to become more toxic at higher temperatures [72].

Discrepant results obtained for the absolute KD60 or M24 values measured between facilities for the same ITN sample is likely to be due to random errors and/or systematic bias in studies. Similar differences have been observed in other multi-centre studies to compare three test methods in determining the bioefficacy of the same nets [35]. Some of the observed differences are likely to be due to testing conditions, procedures, and the different mosquito strains at the two facilities. Differences that cannot be ruled out are temperature which is known to impact mortality [55, 56]. The temperature in PNG was 28 ± 4 °C compared to 27 ± 1 °C at IHI, although control mortality was acceptable at both sites. Variability in cone bioassay procedures i.e. the angle of cone was 45° [46] as per WHO guidelines in PNGIMR, while in IHI the cone test is performed at 60° in the cone assay board to maximize mosquito contact with ITNs although this has been shown to be inconsequential [40]. Net pieces were shipped to Tanzania from PNG by courier in an insulated package with a very short transit time. As such it is unlikely that transport would have affected their bioefficacy. Operator skill may have contributed to the variability of results, but it should be noted that cone bioassays conducted on the same pieces at different time points gave similar results. The *An. gambiae s.s* and *An. farauti s.s* strains used were fully susceptible to pyrethroid insecticides, of a similar age, and exposed to similar colony maintenance conditions; these strains are not sibling species and they have differing morphology [73]. The *An. gambiae* strain used for this test has shown high mortality in WHO cone bioassays against several ITN brands including the ones tested in this current study [74, 75]. Mosquito strain variability e.g., size and fitness may explain some of the variation in absolute values measured. Both strains were assessed for insecticide resistance at the time of testing. Both strains were fully susceptible to pyrethroids at 1 × WHO diagnostic concentration [76], but it is likely that the concentrations needed to knock down but not kill *An. gambiae s.s.* (Ifakara) are lower than the 1 × diagnostic concentration. It is currently unclear how the choice of susceptible laboratory-reared mosquito strains affects the outcomes of WHO cone bioassays and more research is needed to establish robust parameters for comparison. Even so, the WHO cone bioassays in the present study showed very good agreement for nets that demonstrated the highest M24. Unsurprisingly, more variation in results was observed between the testing facilities for ITNs with low KD60 or M24. This is a well-known phenomenon and for this reason, large sample sizes (30–50 nets) are recommended for cone testing used for bioefficacy monitoring of field used ITNs that generally have reduced M24 [8].

In the present study, five of the twenty unused ITNs effectively killed mosquitoes (≥ 80% M24) at IHI. The average M24 measured in cone bioassay (mean from the two sites) for the best and the worst performing unused ITNs was 99% and 24% (Additional file 2), respectively. These results agree well with other studies and WHO specification reports [16, 35, 43–46]. Even so, most ITNs tested at IHI gave higher KD60 than M24. It has been observed that with *An. gambiae* to achieve 80% M24 requires at least a 5% higher net surface concentration of pyrethroid than to achieve 95% KD60 [13]. In a WHOPES report (2008) it was found that for *An. gambiae* the KD60 criterion is met at dosages lower than the M24 criterion, so that 95% KD60 corresponds to 20–30% M24 [31]. It was reported in an expert review that new unused deltamethrin coated nets demonstrate 100% KD60 but 55% M24 [16]. It may, therefore, be inferred that M24 is the more conservative endpoint of pyrethroid performance in a cone bioassay. Indeed, it was previously stipulated by WHOPES that “As the two existing WHO criteria for biological effect in the cone test correspond to different surface concentrations of the active ingredient, they are not equivalent, and one of them should be designated as the basis for WHO specifications. Possibly the criterion could be chosen on a case-by-case basis but mortality is clearly more stringent than KD and therefore appears to be the criterion of choice” [31]. The data from this study corroborate this, and mortality was the more stringent criterium in this work. All analyses conducted in the present study showed greater agreement between the two sites when M24 was used as the endpoint. Spearman correlation showed a very strong correlation of efficacy results for M24 (r = 0.9) between the two testing facilities and the Bland Altman showed more consistent agreement on this endpoint. These results further corroborate other confirmatory analyses of PNGIMR bioefficacy tests conducted at Liverpool School of Tropical Medicine (LSTM) where susceptible *An. gambiae s.s.* mortality estimates were strongly correlated with PNGIMR results (coefficient of determination equal to 0.80) [46]. Five of 20 (25%) of nets had discrepant results between IHI and PNGIMR testing facilities. The number of discrepant results is further decreased if assay-inherent stochastic variability is considered. It should be noted that analysis in this study observed differences in ITN bioefficacy when considering individual net pieces. Because each net piece has only 4 cones there is even greater heterogeneity for comparisons of net pieces. Due to the lower number of replicates the 95% CI of the proportion for the 80% M24 is 58%-93% and for KD60 75%—100%. It is therefore necessary to consider comparison of the combined pieces for each ITN that have a total of 20 replicates each to give a more precise estimation of bioefficacy. There are variations in spatial presentation and/or distribution of active ingredients within the netting, or the surface treatment. This is well recognised as the WHOPES report states “a consequence of the narrow dose ranges over which biological responses change dramatically is that responses cut-off values for decision-making are inevitably set within a region in which small errors in measurements can have a disproportionately large impact. This problem is compounded by the high sampling error associated with the very variable active ingredient distribution in many types of insecticidal netting” [31]. The current work corroborates this statement and for this reason the use of confidence intervals that reflect the natural variability in the bioassay based on the number of replicates used for evaluation is a useful addition to thresholds for bioefficacy criteria. Furthermore, the assay inherent uncertainty should be better accounted for. In this study we used a simple method based on estimates of the 95% CIs around the WHO thresholds of M24 and KD60 when 20 cones i.e., n = 100 mosquitoes are used. Nets with WHO cone bioassay results that fell within this margin of assay-inherent error were still considered as passed. However, precision of the pass/fail could also be improved by increasing sample size and this study suggests that larger sample sizes for QA testing are appropriate.

In this study, however, all nets passed tunnel tests, possibly because of longer mosquitoes exposure time (12 h) compared to the cone assay test (3 min) as well as sugar starvation in the tunnel test [8], which gives higher efficacy even at lower pyrethroid concentrations [21, 77]. Given that it provides the least stringent evaluation and requires the most complex setup, the need for tunnel tests for testing pyrethroid ITN bioefficacy is questionable and may be a means for ITNs with lower surface concentrations of insecticide to pass WHO bioefficacy criteria.

In the present study, four pyrethroid ITN brands were included (Additional file 3). All of these brands had passed WHOPES testing and were recommended (now pre-qualified) based on WHO cone bioassay data. Several brands were selected to increase the generalizability of the findings. Our literature review highlighted that all these brands had passed bioefficacy criteria in the WHO cone bioassays in multiple studies (Table 4). The results from this study agreed with the results of several studies of PermaNet^®^2012 [18, 19, 48, 78, 79], and PermaNet^®^2019 nets [44, 46]. The Yorkool^®^ nets results are similar to WHO prequalification reports [16, 35] and recent results from durability studies in Benin [43] and Madagascar [45]. For the Interceptor^®^ and SafeNet^®^ nets, cone bioassay results in this study were lower than that seen in other studies [15, 20, 23, 35, 80]. Some variability in the surface bioavailability of pyrethroids on ITN samples may be introduced by the manufacturing process [18, 47, 48], variations in spatial presentation and/or distribution of active ingredient within the netting. Net surface bioavailability of pyrethroids can also be affected by insecticide migration rate [31], poor storage or shipping conditions [12] and the binder used [46]. However, we consider it a minimum standard for ITNs to have adequate surface concentration to kill pyrethroid susceptible mosquitoes when they are new.

In this study, the literature review showed that in some countries with a high malaria burden, e.g. Nigeria, ITN QA using a WHO cone bioassays was introduced after a long period of importation of nets with low bioefficacy [81]. It is important for ITN bioefficacy to be evaluated post shipment to ensure that nets procure will perform as required. Acceptable performance of ITNs is defined by WHO as retention of biological activity (e.g. M24 ≥ 80%) through 20 standard washes (or 3 years of use) but there is no simple physiochemical measurement corresponding to this definition [31]. It is generally agreed that a validated, low-cost, easy-to-implement laboratory methodology for assessing surface AI content is urgently needed [30] but current methods have not been found to correspond well to bioefficacy results [34]. WHO cone bioassays have been demonstrated to be highly sensitive to changes of active ingredient concentration on the net surface and could thus play a crucial role in ITN QA [35]. However, many endemic countries do not have well-established cone bioassays for ITN QA (either as post-delivery or pre-distribution QA). WHO cone bioassays were recommended for QA of conventionally treated nets [36, 37]. The tenth WHOPES meeting recommended a WHO standard bioassay to be used for ITN QA purposes until an alternative was developed [35]. The eleventh WHOPES meeting, however, concluded that “WHO standard bioassays cannot be used throughout the world for ITN QA purposes, so physicochemical tests must be used instead” following reasons that WHO cone bioassay outcomes showed variation and were mosquito strain- dependent [31]. The current work adds weight to the argument that the choice of the mosquito strain or differences between laboratories systematically affects the WHO cone bioassays results. We show that WHO cone bioassays are reproducible if the systematic bias is accounted for. This can easily be achieved by conducting studies such as this one but requires partnership between testing facilities and flexibility from policymakers. Further harmonisation of laboratory methods may also assist in minimising inter-facility differences in results. More evidence is needed to test whether M24 criteria should be mosquito species specific (although it should be noted that some nets achieved > 80% mortality with both strains). This can be likened to the already existing species-specific guidance on discriminatory insecticide concentrations used in WHO tube bioassays [76]. Therefore, well-controlled bioassays can be used for QA purposes if there is a will to address the complex realities.

The recent landscape bioefficacy report [30] and several other studies [7, 33, 82, 83] have highlighted the need for better QA. Almost all the studies found in the literature review showed high KD60 and M24 of unused pyrethroid ITNs with pyrethroid susceptible strains. While it could be that there is a bias toward the publication of positive trials (Table 4), the inclusion of the WHOPES reports, and several independent operational monitoring studies suggests that this is not the case. A few independent operational monitoring reports revealed that ITNs that did not pass bioefficacy thresholds were distributed to the endemic population however, these nets had passed the prequalification process with demonstrated high bioefficacy. The authors feel that it is critical that WHO resumes reporting ITN performance data in prequalification reports to be used as a product performance reference by procurement agencies, National Malaria Control Programmes (NMCP), or other bodies that monitor product performance at a country level.

## Study limitations

The number of nets tested may not be sufficient to generalize the study results. Therefore, a literature review on the utility of cone bioassays for unused pyrethroid ITNs testing was also conducted and showed results in broad agreement with those reported here. Many publications and reports included in the literature review did not indicate country of manufacture, ITN age, and the lot or batch numbers of the tested nets, or data collection period. Thus, it was only possible to present the date/year that the study was conducted and/or the publication date. This study was not conducted using the ideal full factorial design with the same strains in each laboratory (that would tease out species versus laboratory differences) due to biosafety concerns because both laboratories are in malaria endemic areas. It would not be safe to establish either malaria vector in the other laboratory.

## Conclusions

Based on these study findings, the WHO cone bioassay is a reproducible bioassay provided inherent stochastic variation and systematic bias are accounted for and agree well where nets pass WHO M24 thresholds. The literature review included in this study confirms that WHO cone bioassay bioefficacy criteria have been previously achieved by all pyrethroid ITNs (unwashed), without the need for additional tunnel tests. The 80% M24 threshold remains the most reliable indicator of pyrethroid ITN quality among pyrethroid susceptible mosquitoes.

## Supplementary Information

**Additional file 1.** Details for tested insecticide-treated nets.

**Additional file 2.** Cone bioassays data per subsamples.

**Additional file 3.** Cone bioassays data per net.

## Data Availability

The data set for this study is available on request from VCPTU.

## Abbreviations

AI: Active ingredient
BFI: Blood Feeding Inhibition
CI: Confidence interval
IHI: Ifakara Health Institute
IMR: Institute of Medical Research
ITNs: Insecticide treated nets
KD60: Knockdown after 60-min
M24: Mortality at 24-h
PNG: Papua New Guinea
QA: Quality Assurance
VCPTU: Vector Control Product Testing Unit
WHO: World Health Organization
WHOPES: World Health Organization Pesticide Evaluation Scheme
